# Early Drug Development and Evaluation of Putative Antitubercular Compounds in the -Omics Era

**DOI:** 10.3389/fmicb.2020.618168

**Published:** 2021-02-02

**Authors:** Alina Minias, Lidia Żukowska, Ewelina Lechowicz, Filip Gąsior, Agnieszka Knast, Sabina Podlewska, Daria Zygała, Jarosław Dziadek

**Affiliations:** ^1^Laboratory of Genetics and Physiology of Mycobacterium, Institute of Medical Biology, Polish Academy of Sciences, Lodz, Poland; ^2^BioMedChem Doctoral School of the University of Lodz and the Institutes of the Polish Academy of Sciences in Lodz, Lodz, Poland; ^3^Institute of Microbiology, Biotechnology and Immunology, Faculty of Biology and Environmental Protection, University of Lodz, Lodz, Poland; ^4^Institute of Molecular and Industrial Biotechnology, Faculty of Biotechnology and Food Sciences, Lodz University of Technology, Lodz, Poland; ^5^Department of Technology and Biotechnology of Drugs, Jagiellonian University Medical College, Krakow, Poland; ^6^Maj Institute of Pharmacology, Polish Academy of Sciences, Krakow, Poland

**Keywords:** *Mycobacterium*, tuberculosis, proteomics, DNA sequencing, transcriptomics, mutagenesis, drug evaluation, drug identification pipeline

## Abstract

Tuberculosis (TB) is an infectious disease caused by the bacterium *Mycobacterium tuberculosis*. According to the WHO, the disease is one of the top 10 causes of death of people worldwide. *Mycobacterium tuberculosis* is an intracellular pathogen with an unusually thick, waxy cell wall and a complex life cycle. These factors, combined with *M. tuberculosis* ability to enter prolonged periods of latency, make the bacterium very difficult to eradicate. The standard treatment of TB requires 6–20months, depending on the drug susceptibility of the infecting strain. The need to take cocktails of antibiotics to treat tuberculosis effectively and the emergence of drug-resistant strains prompts the need to search for new antitubercular compounds. This review provides a perspective on how modern -omic technologies facilitate the drug discovery process for tuberculosis treatment. We discuss how methods of DNA and RNA sequencing, proteomics, and genetic manipulation of organisms increase our understanding of mechanisms of action of antibiotics and allow the evaluation of drugs. We explore the utility of mathematical modeling and modern computational analysis for the drug discovery process. Finally, we summarize how -omic technologies contribute to our understanding of the emergence of drug resistance.

## Introduction

Tuberculosis (TB) is an infectious disease caused by *Mycobacterium tuberculosis*. The disease is one of the top 10 causes of death of people, according to the WHO. Each year, about 10 million people fall ill with TB, and 1.5 million people die. WHO estimates that approximately a quarter of the world population is infected with *M. tuberculosis*, and 5–10% of people will develop active TB during their lifetime. Incidence rates are reported in all countries and age groups. The disease affects mostly men (57%). Women account for 32% of cases. About 11% of cases are children under 15years of age. People with weakened immune systems are at higher risk of developing the disease, with particular emphasis on people infected with HIV. They are about 19 times more prone to TB. Further factors influencing TB’s risk are malnutrition, diabetes, and smoking (World Health Organization, 2019).

*Mycobacterium tuberculosis* is an intracellular pathogen with an unusually thick, waxy cell wall and a complex life cycle. The bacteria are transmitted by aerosol droplets and most often infect the lungs. Generally, *M. tuberculosis* infects alveolar macrophages, but it can also infect other respiratory system cells. The disease may also be extrapulmonary. *Mycobacterium tuberculosis* can infect cells of bones, genitourinary tract, skin, joints, and meninges (Lee, 2015). Mycobacteria invade macrophages and settle the infection by blocking the maturation of phagosomes. The infection of macrophages results in the host response, where various types of immune cells infiltrate the infection site. The influx of immune cells may result in the eradication of the bacteria. Incomplete eradication of bacteria progresses the disease to the latent stage. *Mycobacterium tuberculosis* becomes enclosed in compact and sometimes calcified cell aggregates. *Mycobacterium tuberculosis* slows down its metabolism due to the restriction of the influx of nutrients and oxygen. The disease becomes latent. *Mycobacterium tuberculosis* can persist in the infected individual’s lungs for decades. When the immunity of the person wanes, granulomas liquefy, and bacteria reactivate to the active phase of the disease. The complex life cycle, intracellular life niche, thick cell wall, and the ability to enter prolonged periods of latency make *M. tuberculosis* very difficult to eradicate.

### Current Antitubercular Chemotherapy

The standard treatment of tuberculosis requires 6–20months, depending on the drug susceptibility of the infecting strain. Antibiotics must be taken in combination, as administering a single antibiotic quickly results in pathogen drug resistance. There are four first-line drugs against tuberculosis (isoniazid – INH, rifampicin – RMP, ethambutol – EMB, pyrazinamide – PZA) and nearly 20s-line drugs, which can be administered during the treatment of drug-resistant tuberculosis. The numbers of multidrug-resistant (MDR-TB) and extensively drug-resistant (XDR-TB) strains of *M. tuberculosis* are a major problem for current antitubercular therapy. MDR-TB is resistant to two first-line drugs, RMP and IHN. XDR-TB is resistant to four core antitubercular drugs, followed by resistance to capreofluoroquinolones and one of the three injectable second-line drugs, e.g., amikacin, capreomycin (CM), or kanamycin (KAN). Nearly 484,000 cases of MDR cases of tuberculosis are estimated to exist worldwide. A total of 13,068 cases of XDR-TB were reported by 81 countries, of which most of them were from the WHO European Region and the South-East Asia Region. Detection of MDR-TB first requires confirmation of TB, followed by testing for drug resistance. In 2018, 51% of people with bacteriologically confirmed TB were tested for RMP resistance (up from 41% in 2017). Even though between 2017 and 2018, there was progress in testing, detection, and treatment of MDR-TB, only 56% of MDR cases were successfully treated globally and only 39% of cases of XDR-TB. As of 2020, WHO recommends that MDR-TB patients are to be treated with fully oral drug regimens. Injectable agents should only be used if other options are not possible. Two such agents, KAN and CM, are no longer recommended (World Health Organization, 2019). High numbers of TB patients, including patients infected with drug-resistant *M. tuberculosis* justify the need to search new antitubercular compounds that could be introduced to antitubercular chemotherapy.

### Prospective Antitubercular Chemotherapy

**Figure 1:**
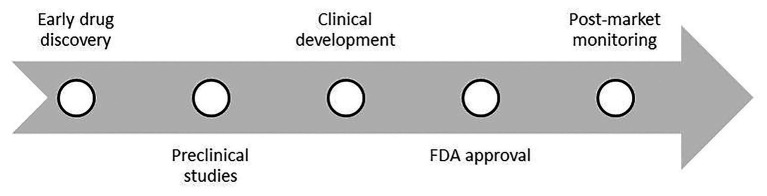
The schematic overview of the drug discovery process.

For a long time, the development of XDR-TB left patients without further options for treatment. The principal drugs for the treatment of tuberculosis were discovered between the 1940s and 1970s (streptomycin, para-aminosalicylic acid, INH, cycloserine – CS, KAN, RMP, and others; Murray et al., 2015). The path of drug discovery is long and costly (Figure 1). The process starts with the early drug discovery stage. Here, researchers identify potential inhibitors in laboratory conditions and assess their principal biological impact. The next stage is preclinical studies. This time chemicals are tested not only on bacteria but also on cell lines or live animals. The knowledge gained in this phase is helpful in Phase III of the study, which considers the drug doses tested here in later human studies. Several experimental tools are used in the preclinical stage. One is the *in vitro* hollow-fiber system that provides data to improve animal experimentation. The great advantage is the integration of this data with data from many different animal models. Such models are the well-known BALB/c mice and the newer Kramnik mouse model, or the marmoset and rabbit models. The preclinical stage provides valuable information about the activity of the tested drug or sterilization of the pathogen. Unfortunately, it provides limited information on the pharmacokinetics and pharmacodynamics of the drug (Dooley et al., 2019).

The main cause of failures in the clinical development of drugs is its insufficient effectiveness, which is associated with an accurate determination of pharmacokinetics (Muliaditan et al., 2017). Before entering clinical trials, it is essential to gather much information about the exposure-response relationship and the relationship between pharmacokinetics and toxicity of the tested substance. Phase I clinical trials provide information on pharmacokinetics and safety, an important element here is to establish drug interactions with food intake or whether the dose depends on the patient’s body weight. Phase II studies provide detailed inter-population information. In Phase III, drug efficacy is assessed by collecting post-drug exposure data, timed microbial response, and safety (Dooley et al., 2019). After clinical trials, the development of a new drug requires approval by the Food and Drug Administration (FDA).

In 2012, the United States FDA approved the use of a novel antitubercular drug bedaquiline – BDQ. BDQ was discovered by the pharmaceutical company Johnson and Johnson, under the brand name is Sirturo (Deoghare, 2013). It is the first member of a new class of drugs called diarylquinolines. BDQ is a bactericidal drug. BDQ blocks proton pump of ATP synthase, encoded by gene *atpE*. ATP production is essential for cellular energy turnout (Koul et al., 2008; Deoghare, 2013). BDQ is recommended strictly for the treatment of MDR-TB, and when options to treat this condition using existing drugs have been exhausted. BDQ should not be used to treat latent TB infection. It should not be used alone but as part of combination therapy and never added alone to a failing regimen. By the end of 2018, 90 countries reported having imported or using BDQ (World Health Organization, 2019).

In 2014, European Medicine Agency (EMA) conditionally approved a second novel antitubercular medicine, delamanid (DLM), as a part of combination therapy to treat adults with MDR-TB. The brand name of DLM is Deltyba. DLM exhibits a low minimum inhibitory concentration, distinguishing itself from other clinically approved drugs. This medicine is a pro-drug that requires metabolic activation for its action. It is activated by the deazaflavin F420-dependent nitroreductase. Resistance against DLM includes mutations in genes participating in pro-drug activation or associated with the cofactor Ddn biosynthetic pathway. DLM is known for specifically inhibiting the synthesis of two mycolic acids – keto mycolic acid and methoxy mycolic acid. They are the building components of the mycobacterial cell wall (absent in Gram-positive or Gram-negative bacteria). These components are also making it difficult for medicines to penetrate the cells. The use of DLM allows for more effective treatment through disrupting cell wall and shortening a treatment regimen (Tiberi et al., 2018b; Bahuguna and Rawat, 2020). By the end of 2018, 57 countries reported having imported or started using DLM (World Health Organization, 2019).

Approval of new medicine in Phase III clinical trials is always a risk, but benefits are perceived as more significant. BDQ was the first member of a new class of medicines. The principal study showed that treatment with Situro was effective; the drug worked well and fast. BDQ and DLM are well-working antibiotics, but there is already resistance against BDQ. In particular, they are mutations in *atpE*, gene coding ATP synthase subunit c, the target of BDQ, or gene Rv0678, which plays a role in regulating the expression of the MmpS5-MmpL5 efflux pump (Nguyen et al., 2018; Ghajavand et al., 2019). New antibiotics are most effective in a few first years before resistance is developed and disseminated across the bacterial population. Therefore it is vital to search for new medicines. As of October 2020, a few antitubercular drugs are currently in phase III or II clinical trials (Table 1). In addition to new drugs, there are also repurposed drugs like clofazimine (CFM), levofloxacin (LFX), moxifloxacin (MFX), and linezolid (LZD), which are in phase II and phase III trials for TB too. Drugs are also tested for repurposing from the treatment of other diseases, auranofin that is an antirheumatic agent and nitazoxanide that is an antiprotozoal agent. The growing TB epidemic again developed an interest in CFM, which now is an important constituent of newer TB regimens. CFM is a pro-drug, but the exact mechanism of action is not yet known. MFX is investigated in regimens combining BDQ, pretomanid, and PZA, or rifapentine (Bahuguna and Rawat, 2020).

**Table 1:** New drugs in phase III and II clinical trials (Vjecha et al., 2018; Bahuguna and Rawat, 2020).

Drug	Chemical class	Target	Effect	Clinical status
Bedaquiline	Diarylquinoline	ATP synthase	Inhibits energy metabolism of the cell	Phase III^*^
Delamanid	Nitroimidazole	Exact target not yet known	Inhibits mycolic acid synthesis (keto and methoxy mycolic acids) and cell respiration	Phase III^*^
Pretomanid	Nitroimidazole	Exact target not yet known	Inhibition of cell wall synthesis and respiratory poisoning	Phase III
Delpazolid	Oxazolidinone	50S subunit of the ribosome	Inhibits protein synthesis	Phase II
Sutezolid	Oxazolidinone	50S subunit of the ribosome	Inhibits protein synthesis	Phase II
SQ109	Diamine	MmpL3	Inhibits cell wall synthesis	Phase II
Macozinone (PBTZ169)	Benzothiazinone	DprE1	Inhibits cell wall synthesis	Phase II
Telacebec (Q203)	Imidazopyridine	Cytochrome *bc1* complex	Inhibits ATP synthesis	Phase II

* Recently approved drugs.

### Host-Directed Therapy

Treatment of *M. tuberculosis* infection with currently available antibiotics has several negative features associated with drug toxicity, and the problem is the increasing presence of drug-resistant *M. tuberculosis* strains. Another approach to supporting the treatment and prevention of tuberculosis is host-directed therapy (HDT). This strategy aims to modify host response related to the development, activity, and pathogenicity of *M. tuberculosis* infection. The HDT agents may have immunomodulatory properties, enhance the host’s immune system or influence the host’s metabolic pathways, which should aid in fighting the pathogen and protect the lung tissue (Tiberi et al., 2018a). One way is to activate autophagy, which would contribute to the increased intracellular killing of *M. tuberculosis*. In this case, the possibility of using rapamycin, metformin, statins, vitamin D, phenylbutyrate, carbamazepine, or valproic acid is investigated. Rapamycin inhibits the activity of mammalian target of rapamycin (mTOR), which is an inhibitor of autophagy. However, the use of this compound in therapy is limited due to the potential for side effects and its breakdown by the liver enzyme CYP3A4, which is activated by RMP, one of the first-line drugs in the treatment of tuberculosis. In addition, an increase in replication was observed in cells co-infected with HIV and H37Rv in response to rapamycin. Another potential compound is metformin, which can increase AMP-activated protein kinase and reactive oxygen species expression. These abilities contribute to activating autophagy and reducing inflammation. Statins lower lipid levels by inhibiting the enzyme *β*-hydroxy β-methylglutaryl-CoA, which is involved in lipid metabolism. Statins have a positive effect on the maturation of phagosomes and autophagy processes and reduce the accumulation of lipids inside cells, e.g., in macrophages, limiting the growth of the pathogen. Vitamin D and phenylbutyrate may increase the expression of LL-37, cathelicidin. Moreover, vitamin D regulates the expression of cytokines and immune mediators. Carbamazepine and valpronic acid are responsible for the activation of mTOR-independent autophagy (Dara et al., 2019; Torfs et al., 2019; Ahmed et al., 2020).

Host-directed therapy may also target the disintegration of the granuloma structure. Etanercept, an inhibitor of tumor necrosis factor *α* (TNF-α) involved in the formation and maintenance of granuloma, may help treat tuberculosis. Another possible drug is bevacizumab targeting vascular endothelial growth factor (VEGF). The drug influences the normalization of the vessels, which in turn causes a change in the morphology of the granuloma and the possibility of interaction with anti-tuberculosis drugs.

An important path of HDT is immunomodulation, increasing the anti-inflammatory response, which would help to reduce tissue damage. Ibuprofen, diclofenac, acetylsalicylic acid, and vitamin D are of interest (Torfs et al., 2019; Ahmed et al., 2020). Nonsteroidal anti-inflammatory drugs such as ibuprofen or diclofenac can reduce the inflammatory response by inhibiting cyclooxygenases. Acetylsalicylic acid activates lipoxin A4, which inhibits neutrophil migration and TNF-*α* production (Torfs et al., 2019; Ahmed et al., 2020; Young et al., 2020).

### Vaccination

Bacillus Calmette-Guérin (BCG) is a vaccine based on attenuated *Mycobacterium bovis*, and it has been available since 1921. The BCG vaccine is currently applied worldwide, mostly in high burden countries of Africa, Asia, and South America. In 2011, among the 180 countries with available data, 157 countries recommended universal BCG vaccination (Zwerling et al., 2011). BCG vaccine efficiency is limited, as reflected by the number of tuberculosis cases worldwide. Therefore there is an ongoing search for novel, more effective vaccines. Several types of novel vaccine candidates are currently in clinical trials. They are composed of recombinant proteins and adjuvants, they are viral vectored, and they are attenuated and whole-cell vaccines (Kaufmann, 2020). The call for new vaccines is supported by the World Health Organization. The principal recommendations are that the new vaccine should be affordable, safe, and more efficient than the current BCG vaccine in the prevention of infection, disease, or recurrence.

One of the most promising vaccine candidates is the M72 subunit vaccine developed by GlaxoSmithKline. The vaccine successfully passed a phase IIb clinical trial. It was 54% effective (Van Der Meeren et al., 2018). The study tested booster vaccination of HIV-positive adults with latent TB infection who had been vaccinated with BCG as infants. This vaccine contains two TB antigens, fused in one protein and combined with AS 01E as an adjuvant. The disadvantage of this adjuvant is its high cost of production and limited availability, which may be an obstacle to the wide scale M72 vaccination (Kaufmann, 2020).

### Antitubercular Drug Development Market

It takes a lot of time and cost to bring a new drug to market. The average cost that pharmaceutical companies have to bear is about US$ 2.6 billion during 10years of research and development. Clinical trials consume most of this funding, about US$ 1–2.5 billion. Clinical trials are also the longest stage during the drug discovery process – they can last up to 6–7years. Funding for the prevention, diagnosis, and treatment of TB has doubled since 2006, but it is still insufficient. In 2019, 119 low- and middle-income countries funding reached US$ 6.8 billion, up from US$ 3.5 billion in 2006. Most funds (about 87%) are available from domestic sources. Pharmaceutical companies mainly research new antibiotics. Most of them are small and medium-sized institutions (81%). Academia carries out 12% of new antibiotic research. Large pharma companies account for 3% of research. Non-profit institutions and public-private partnerships carry out the rest (Theuretzbacher et al., 2020). In 2019, international donor funding amounted to US$ 0,9 billion, which is far below than what was assumed by the Stop TB Partnership’s Global Plan. Most of the international donor funding comes from the Global Fund to Fight AIDS, Tuberculosis, and Malaria. According to data from Treatment Action Group, there was the funding of US$ 772 million for TB research and development in 2017, which is much less than the target of at least US$ 2 billion per year set at the UN high-level meeting on TB (World Health Organization, 2019). High costs born during the drug development process justify the introduction of new technological solutions, including -omic technologies, that can facilitate the introduction of new, effective, and safe drugs to the market.

## Strategies to Find new Drugs for Tuberculosis Treatment and the Drug Discovery Process

Designing a new drug and bringing it to market is a very time-consuming process that can take up many years (Hughes et al., 2011). This process is also highly costly, with little prospect of reimbursement from developing countries where tuberculosis is most prevalent. There are several important points to consider when searching for new antitubercular drugs. There is a need to provide shorter, simpler, and affordable multi-drug regimens for drug-sensitive *M. tuberculosis*; shorter, more effective, less toxic, and less expensive regimes for drug-resistant *M. tuberculosis*; and shorter, more straightforward, easily tolerable, and safe regimes for latent tuberculosis. Furthermore, new antitubercular drugs should not antagonize other medications, such as those used during HIV infection treatment. Finally, ideal drugs are those that have restricted the occurrence of drug-resistance.

**Figure 2:**
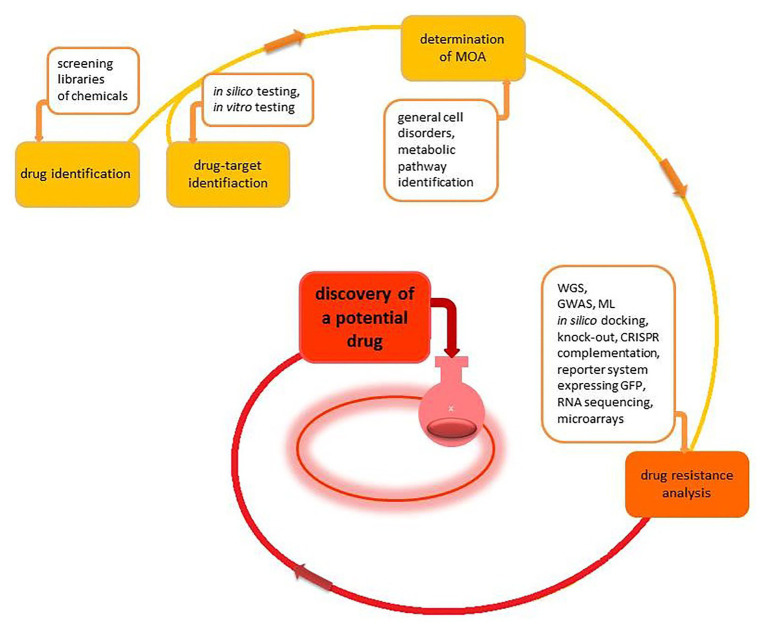
The schematic overview of the early drug discovery process. The process begins with the identification of either a drug target or the drug itself. Next, the determination of the mode of action (MOA) and consideration of drug resistance facilitates an indication of safe and practical potential new drugs.

There are two major paths to discover new antibiotics during the early stage of drug development (Figure 2). The first approach involves screening libraries of chemicals to find a “hit” – a molecule that kills a pathogen at the desired concentration. The advantage of this approach is that bacterial cell growth, compound penetration, and target sensitivity are resolved at the time of identification. Once an active chemical is identified, it is vital to establish several issues to evaluate the utility of the compound properly. The first issue is the identification of the drug target protein. Pinpointing the drug target leads to an explanation of the mode of action (MOA) of the novel drug (Table 2).

**Table 2:** Approaches to establishing MOA of new drugs.

Aim	Methods	Example studies
Identification of general cell disorders and disrupted pathways and linking them to the disrupted metabolic pathway	RNA-Seq	O’Rourke et al., 2020
	Analysis of metabolites by LC-MS	Halouska et al., 2012
	Analysis of lipid content by LC-MS	Pal et al., 2018
Identification and confirmation of the drug target	WGS to identify mutations in the drug target	Andries et al., 2005
	Generation of knock-out strains	Bryk et al., 2008
	Generation of complemented mutants with increased gene expression	Kumar et al., 2015
	Generation of complemented mutants with decreased gene expression	Esposito et al., 2017
	CRISPR system gene expression depletion	McNeil and Cook, 2019

Further, it facilitates the optimization of the chemical structure of the drug. Finally, identifying the drug target protein provides essential information regarding possible causes of potential drug resistance. It is also pertinent to establish if the drug generates adverse effects, like an increase in the pathogen virulence. Finally, the amount of drug resistance variants and drug resistance sources should be determined (Table 3). Following the evaluation of the influence of the drug on the bacterium, the chemical toxicity is tested against eukaryotic cells. If the chemical is toxic at low doses for the pathogen but not toxic for eukaryotic cells, the compound may be further tested for its effectiveness in intracellular infection models and animal models before reaching the clinical trial phase.

**Table 3:** Identification of sources of drug resistance.

Aim	Methods	Example studies
Identification of the modification of the drug target	WGS of the resistant strains	Takiff et al., 1994
	GWAS	Farhat et al., 2019
	*In silico* docking	Nachappa et al., 2020
Identification of the modification of the disrupted metabolite pathway	RNA-Seq	Reviewed in Briffotaux et al. (2019)
	TraSH	Viswanathan et al., 2017
	PIP networks	Raman and Chandra, 2008
	GWAS and ML	Yang et al., 2018; Deelder et al., 2019
Induction of the efflux pumps	Using reporter system expressing fluorescent protein	Jain et al., 2016
	RNA-Seq	Reviewed in Briffotaux et al. (2019)

The second approach for finding new antibiotics begins with identifying a molecular target that is essential or otherwise important for the pathogen virulence. The proteins that make good targets for antibiotics are those for which mutations are often deleterious. Such an approach makes them less susceptible to the random development of resistance. Inhibitors of those proteins can be found in two ways: through *in silico* screening using bioinformatic analyses and tested in experimental conditions (Korycka-Machala et al., 2017) or through *in vitro* screening inhibitor trough enzymatic/colorimetric assay (Syre et al., 2003; Grzelak et al., 2019). Potential molecular targets proposed in previous studies are, for example, proteins associated with DNA repair systems (for review, see Minias et al., 2019), DNA replication (for review, see Płocinska et al., 2017), multi-drug efflux pumps (Viveiros et al., 2003; Balganesh et al., 2012), or proteins necessary for cell division (Plocinska et al., 2012; Chatterjee et al., 2018; Gorla et al., 2018).

The ongoing digital revolution introduces novel solutions to old problems. New bioinformatic technologies and the availability of these advanced technologies for research allow for significant advances in the field of drug development. This review provides a perspective on how modern -omic technologies facilitate the drug discovery process for tuberculosis treatment. We discuss how methods of DNA and RNA sequencing, proteomics, and genetic manipulation of organisms increase our understanding of mechanisms of action of antibiotics and allow the evaluation of drugs. We explore the utility of mathematical modeling and modern computational analysis for the drug discovery process. Finally, we summarize how -omic technologies contribute to our understanding of the emergence of drug resistance.

## Identification of Antibacterial Compounds

### Screening Trough Libraries of Chemicals

Antibacterial compounds can be identified in high-throughput screens (HTS), which involve searching through a library of chemicals against either bacterial cell culture or chosen bacterial proteins in *in vitro* assay. When there is an observable inhibition of growth or enzyme activity at the desired concentration, the compound is tested further. The key to every HTS endeavor is the compound collection. Libraries of compounds are designed and selected for drug-like properties and structural diversity, critical to identifying unique hits for screening targets (Lushington and Chaguturu, 2014). The main challenge in this approach is the quality of chemical libraries. When creating a library, it is important to pay attention to its composition by excluding compounds that may interfere with screening results. Libraries should not contain unstable, highly reactive, or insoluble compounds. Assessment of the identity of the relationship and validation of purity is also essential. It is important to develop the standard of libraries by engaging library creators in their development, and also scientists dealing with screening research and commercial library managers. There is a forum (Nature Chemical Biology) that brings together this community, where issues in this field are presented and standards are discussed (Screening we can believe in, 2009). Millions of compounds are now commercially available, which allows for the development of research for both academics and the pharmaceutical industry.

### Adjusting Culture Conditions

Bacterial culture can be carried out in various environmental conditions. Researchers often try to adapt and resemble culture conditions to those inside the human cells during pathogen infection. Screening of compounds can be done with reporter assays using different types of culture. Grant et al. (2013) screened a library of compounds against both actively replicating and non-replicating bacilli. They constructed fluorescent reporter assays for replicating and non-replicating conditions. Screen of compounds with both assays allowed to characterize the compounds as having effect on only replicating activity, only non-replicating activity, or both replicating and non-replicating activity.

### Using Surrogate Models

Working with *M. tuberculosis* brings many difficulties. For example, it is a slow-growing strain, requiring a BSL-3 (biosafety level) laboratory. Such aspects limit the possibilities of seeking new drug targets. A solution is the use of surrogate strain – *Mycobacterium* (*Mycolicibacterium*) *smegmatis*. It grows faster than *M. tuberculosis*, and working with it is more safe. *Mycobacterium smegmatis* shows good compliance with antitubercular drugs if it is grown in a low nutrient culture medium (Lelovic et al., 2020). The study by Altaf et al. (2010) involving screening of chemical libraries, showed that around 50% of inhibitors active against *M. smegmatis* are also active against *M. tuberculosis*. Another surrogate bacterium, slow-growing *M. bovis* BCG, got much better results with the majority of correlating hits. The less common surrogate models used in the antitubercular drug development process are *Mycobacterium aurum* (Gupta and Bhakta, 2012) and *Mycobacterium marinum* (Boot et al., 2018).

## Identification of the Drug Target and Determination of its Mode of Action

As the mechanism of action of individual antibiotics was discovered, it was understood that each of these drugs had a specific target. In addition to inhibiting their primary target, many drugs affect cell metabolism by generating toxic intermediates and triggering a cascade of molecular events, resulting in significant cell changes. Therefore, it is vital to consider the overall cellular metabolism when the cell is under the influence of the antibiotic (Kana et al., 2014).

### Whole-Genome Sequencing

One of the principal paths to identify the drug target is to look for drug resistance mutations, as they often occur in the target. This can be done by whole-genome sequencing (WGS) of the resistant strain. Once the mutation is identified, it should be confirmed by the generation of drug resistant mutant after the introduction of the mutation into a drug-susceptible strain. This approach was used for target identification of BDQ. The authors generated drug-resistant variants of *M. smegmatis* and *M. tuberculosis* and sequenced their genomes. They found that the mutations in *atp*E gene are responsible for resistance to the compound. The *M. smegmatis* wild-type (WT) strain was transformed with a construct expressing the ATP synthetase subunit of the *M. smegmatis* mutant. The complementation with the mutant allele caused drug resistance (Andries et al., 2005). Recently, using a similar strategy, we identified a drug target for 1H-benzoimidazole derivatives. WGS identified mutations in the *mmpl3* gene encoding the integral membrane protein. Strains with trans-complementation of the wild-type mutated target gene were prepared. The resulting 1H-benzoimidazole resistance confirmed the role of the gene in the resistant phenotype (Korycka-Machała et al., 2019).

### Genetic Modification of Mycobacteria

The information regarding the genomic DNA sequence provides a base for genetic manipulations. Several genetic modification approaches are currently available, including the construction of knock-out mutants, complemented mutants, and the use of reporter systems and interference systems, including the CRISPR/dCas system. Gene replacement by homologous recombination allows obtaining unmarked genetic mutants carrying large deletions within the genes of interest. These mutants can be complemented with genes of interest under native or inducible promoter (Parish and Stoker, 2000). One can also silence the gene using CRISPR/dCas (Choudhary et al., 2015). Obtaining a mutant with regulated target depletion allows performing several experiments. The use of such strain allows assessing the impact of the depletion of the studied gene in various conditions (anaerobic, acid pH, and antibiotics). Previously, CRISPR/dCas mutants were used to analyze the MmpL3 as a therapeutic target. GoldenGate cloning was used to develop the CRISPR/dCas plJT965 plasmid. As a result, a 6-fold decrease in expression of the *mmpL3* gene was obtained. The mutation led to a 5-fold increase in strain sensitivity to Mmpl3 inhibitor (McNeil and Cook, 2019).

Complementation of mutants with possible drug-target genes allows control of gene expression, which can be used for the evaluation of chemical compounds against specific targets. An example of this approach is evaluating the available library of GlaxoSmith Kline compounds as CTP inhibitors of PyrG synthetase. This essential enzyme is involved in several biochemical pathways affecting several aspects of *Mycobacterium* physiology. Compounds were tested against *M. tuberculosis* conditional knockdown strain using a Pip-ON inducible system. A mutant carrying the *pyrG* gene under promoter induced by pristinamycin I was designed. The dependence of the action of two compounds from the analyzed library on the PyrG level confirmed that the enzyme is an intracellular target (Esposito et al., 2017). Similarly, the antitubercular activity of known compounds was confirmed by constructing conditional mutants of the tetracycline-induced *panC* gene, identifying the inhibitory activity of flavonoid derivatives (Abrahams et al., 2012).

To further study the drug target, it is also useful to overexpress the gene of interest. For the analysis of antifolates, an *M. tuberculosis* strain carrying the plasmid pMRN1 containing a wild copy of the *dfrA* gene under the control of the strong promoter was constructed. A 4-fold increase in the MIC 90 strain overexpressing the gene encoding dihydrofolate reductase was observed for 17 compounds, which indicates the targeted nature of these compounds (Kumar et al., 2015). Similarly, *M. smegmatis* strain overexpressing MtNadD was prepared using the non-interfering plasmid pVV16 and the deletion mutant. The strains were tested in the presence of benzimidazolium (N2) derivatives. The overexpressed strain consistently showed a larger MIC compared to the mutant strain (Osterman et al., 2019).

### Transcriptomics

When identifying an antibiotic’s mode of action, one can look for changes in the metabolism, growth, and morphology of bacteria. These observations allow the assessment of bacterial target inhibition through comparison with known mechanisms (Silver, 2012). So an alternative route to identify potential drug targets is through transcriptomic data obtained either through microarrays or RNA-Seq (Fields et al., 2017). O’Rourke et al. (2020) investigated transcriptomic profiles of 37 antibiotics within six different mechanisms of action, which allowed blind predictions of the antibiotic class based on transcriptomic response with an accuracy of <80%. A similar model was developed for *M. marinum* (Boot et al., 2018). Betts et al. (2003) examined *M. tuberculosis* gene expression after treatment with INH, triclosan, and thiolactomycin. Based on gene expression changes, a transcription profile model was proposed that enabled the determination of differences between *M. tuberculosis* treated with each of the three drugs. This model can be used to determine the MOA of uncharacterized mycolic acid biosynthesis inhibitors (Briffotaux et al., 2019). Differentially expressed genes were also evaluated in a whole blood model under the influence of RMP, INH, PZA, and EMB (Kwan et al., 2020). Currently, there are over a hundred reports of differential expression of mycobacterial genes. The results from these reports were recently combined into model INDIGO-MTB. The goal of the model was to identify antibiotic combinations that are most promising for TB drug development. The authors identified the transcription factor Rv1353c as a regulator of multiple drug interaction outcomes. They concluded that this factor could be targeted for rationally enhancing drug synergy (Ma et al., 2019).

### Metabolomics

Metabolomics is a useful approach for finding new targets for antituberculosis drugs and understanding compounds’ mode of action (Tuyiringire et al., 2018). By analyzing the network of metabolites and the interactions between them, it is possible to obtain knowledge about the cell processes (Goff et al., 2020). de Carvalho et al. (2010) supplemented *M. tuberculosis* cultures with 13C-labeled carbon substrates and then analyzed the metabolites by LC-MS, proving that *Mycobacteria* can catabolize multiple carbon sources simultaneously. In the following study, Prosser and Carvahlo used LC-MS to interrogate the antibiotic action mechanism of d-cycloserine (Prosser and de Carvalho, 2013). Halouska et al. (2012) described metabolomics changes in model *M. smegmatis* under the influence of 12 known drugs and three chemical leads. Nuclear magnetic resonance (NMR) analysis of the *M. smegmatis* metabolome clustered drug-induced patterns, correlating them with *in vivo* drug activity. Zampieri et al. (2018) analyzed the metabolomic response of *M. smegmatis* to 62 reference compounds. They used that information to predict the MOA of a library of 212 new anti-mycobacterial compounds from the pharmaceutical company GlaxoSmithKline.

A separate part of metabolomics is lipidomics, which studies the interactions between currently known lipid species and other lipids, proteins, and metabolites in the cell (Wu et al., 2014). It is based on the use of mass spectrometry (MS), a technique by which the mass-to-charge ratio and the number of ions are measured, gas chromatography (GC-MS), and liquid chromatography (LC-MS; Griffiths and Wang, 2009). Analysis of cell lipid content changes in response to changing environmental conditions may lead to the identification of key pathways in lipid biosynthesis. Pal et al. (2018) used lipidomics to show that INH treatment of *M. tuberculosis* can alter the composition of glycerolipids and glycerophospholipids.

## Choosing the Drug Target and Finding its Inhibitors

Because of a significant understanding of the processes taking place in bacterial cells, the number of potential molecular targets for inhibition is very long. Proteins taking action in DNA metabolism and cell wall synthesis are of particular interest. Other attractive targets for new drugs are proteins from the RND family (resistance, nodulation, and cell division), especially Mmpl3, for which analogs of EMB displayed inhibiting activity (Campaniço et al., 2018).

### Genetic Modification of Mycobacteria

The alternative approach used to identify new drugs is first identifying the target and then looking for its inhibitors. It is usually done by creating knock-out mutants, complemented mutants, and/or CRISPR/dCas mutants with specific gene changes. An important aspect to bear in mind is those target proteins identified as essential may be significant for bacterial survival only under laboratory conditions and not in the infection process (Zuniga et al., 2015). Similarly, part of the mutants obtained in laboratory conditions, during growth in laboratory media, is not viable during animal infection. To test the essentiality of individual genes, the researchers infect animals with knock-out mutants. In an exemplary study, guinea pigs were inhaled with a knock-out mutant of *dlaT*, which product is involved in the restriction of nitric oxide-derived reactive nitrogen intermediates. The authors confirmed a vital role of *dlaT* in establishing infection and searched for *dlaT* inhibitors trough screening a library of chemicals (Bryk et al., 2008). A desirable targeting strategy for drugs is to find a target that will shorten treatment duration and reduce the incidence of tuberculosis relapses. Hu et al. analyzed the *M. tuberculosis* HspX protein, which they previously linked to inhibiting this organism’s growth. The BALB/c mice were infected with *hspX* deleted mutant and the WT strain. The animals were treated with popular antibiotics. Treatment of mice infected with the *hspX* mutant resulted in faster clearance of bacteria from internal organs (Hu et al., 2015).

### Transposon Mutagenesis

A high-throughput method of obtaining mutants is transposon site hybridization (TraSH) developed by Sassetti et al. (2003) and DeJesus et al. (2017). The knowledge about gene function comes from inserting transposons at AT sites randomly distributed across the genome into the gene and disrupting its functions. Transposon mutagenesis allowed for a search of novel molecular targets such as virulence factors, enzymes of crucial metabolic pathways, and other essential proteins (Alksne and Dunman, 2008). Transposon mutants can be used to simultaneously analyze a large number of mutants for survival in animal models. Transposon mutants were tested in mice, guinea pigs, and macaques (Sassetti and Rubin, 2003; Hernandez-Abanto et al., 2007; Dutta et al., 2010). In a study by Carey et al. (2018) transposon mutagenesis was used to investigate genetic requirements for the *in vitro* growth of clinical strains of *M. tuberculosis* and the reference *M. tuberculosis* strain H37Rv. The authors identified different requirements for genes in a panel of clinical strains. One of them turned out to be *katG*, encoding the first-line activator of INH.

Originally TraSH was performed using microarrays. Currently, a frequent variant of TraSH is the sequencing of the transposon insertion (Tn-seq). The method is sensitive, and it does not rely on a pre-existing array. It is important to note that transposon mutagenesis is a high-throughput method, and as such, it is affected by a certain level of inaccuracy. The analysis of constructed mutants should confirm results obtained for individual genes.

### Lipidomics

In the studies conducted by Raghunandanan et al. (2019) the authors analyzed the changes in the lipid content of *M. tuberculosis* undergoing hypoxia and subsequent re-oxygenation. It turned out that the dormant bacteria’s lipid content was drastically low and increased during oxygenation. Despite the drastic reduction in lipid synthesis pathways during hypoxia, some of them were still acting. These pathways are potential targets for antituberculosis drugs.

### Bioinformatic Predictions of Drug Targets

Potential drug targets can be identified in protein-protein interaction networks (PIP) studies. Two staples of this method are the identification of the unique non-homologous proteins through the Kyoto Encyclopedia of Gene and Genome (KEGG) database or UniProt and identification of essential genes through the Database of Essential Genes (DEG; Amir et al., 2014; Melak and Gakkhar, 2014, 2015). Search of Amir et al. (2014) for unique proteins of *M. tuberculosis* in metabolic pathways with the KEGG database brought up five pathways consisting of 55 proteins. Selected proteins were analyzed with DEG and UniProt to choose the best candidates for new molecular targets. In another study with a similar approach done by Melak and Gakkhar, out of 1,091 essential genes, 572 were absent in the human genome. The interactome analysis with the STRING database allowed to limiting the number of possible targets. The authors then chose 131 proteins within the close neighborhood of the center of gravity of the proteome network, seeing that they function as important communicators between different metabolic pathways. Most of them were associated with cell wall metabolism. To validate this method, researchers compared their results to known and potential drug targets. Forty-three proteins were already known targets, and some were already reported as candidates (Melak and Gakkhar, 2015). One of the obstacles in this research type is that many *M. tuberculosis* proteins do not have a known function or a 3D structure available. Protein structure is important to conclude the function. However, there are attempts to use proteins with unknown functions (hypothetical proteins) as molecular targets through homology modeling (Uddin et al., 2019). To solve this issue, researchers use their 3D models (Kushwaha and Shakya, 2010) or use The Protein Data Bank (PDB; Melak and Gakkhar, 2015).

### Bioinformatic Predictions of Target Inhibitors

**Figure 3:**
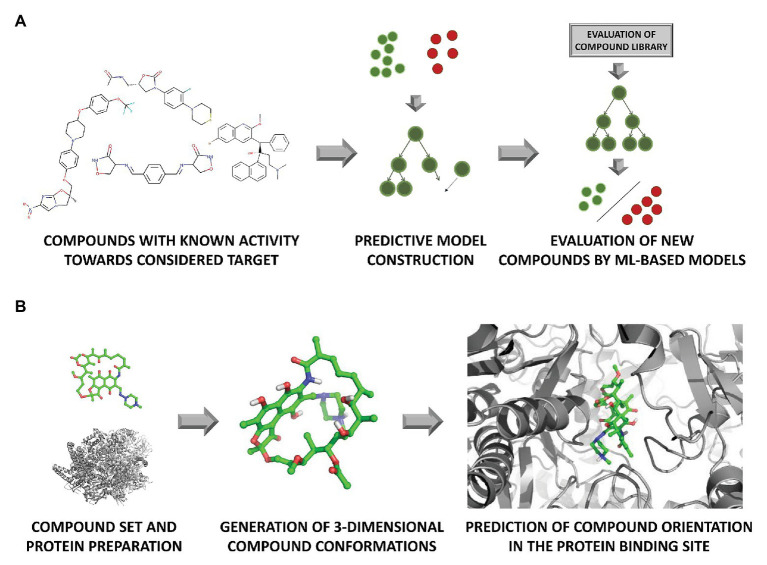
*In silico* evaluation of compound activity with the use of **(A)** machine learning (ML) algorithms representing ligand-based approach, and **(B)** docking (element of the structure-based path).

The number of chemicals that are required for screening in order to find an appropriate inhibitor can be overwhelming. Therefore HTS is often facilitated by virtual screening (VS; Figure 3). VS utilizes computational methods to screen through ligands libraries to find new hits (Kar and Roy, 2013). Molecular docking and pharmacophore modeling are the most commonly used tools (Macalino et al., 2020), being the basis for the distinction of VS approaches into the structure- and ligand-based path. When information about the arrangement of the target atoms is available (e.g., thanks to the presence of the respective crystal structures), it is usually used for docking. Otherwise, the target structure needs to be modeled using homology or *de novo* (restricted to small proteins) modeling (Schmidt et al., 2014). Docking enables rough estimation of compound affinity to the target and making compound comparisons based on the quality of fitting to the binding pocket. Ligand-based VS also uses a quantitative structure-activity relationship (QSAR) analysis for predicting the activity and physicochemical properties of new potential drugs (Nowosielski et al., 2013; Adeniji et al., 2018). Similarity search approaches look for compounds with similar structures to already known ligands, according to the assumption that compounds with similar chemical structures should induce similar biological effects (Martin et al., 2002).

Virtual screening is an useful tool to identify new inhibitors for the key cellular components. For example, VS identified potential inhibitors of MraY, which is necessary for peptidoglycan synthesis (Mallavarapu et al., 2019). Other important cellular components are proteins containing the most frequently occurring drug-resistance mutations like InhA, FabD, and AhpC, which are INH targets. Through the use of VS, researchers can identify new potential drug candidates and limit the number of compounds that need testing in experimental conditions (Jagadeb et al., 2019).

Alternatively, molecular docking aids in finding more effective antitubercular drugs, which structure is based on already existing compounds with proven abilities to inhibit or completely stop *M. tuberculosis* growth. An example of such a study regarding the antitubercular drugs is the search for new inhibitors of arabinosyltransferase C enzyme (EmbC). EmbC participates in the formation of the cell wall, and it is probably the target of EMB. Researchers performed molecular docking of five new derivatives of the EMB. Based on bioinformatic results, the authors indicated two of them that should bind to the EmbC with higher affinity (Das et al., 2020).

Intensive growth of computational power on the one hand, and the increasing amount of data both in the ligand- and structure-based field, has made simple statistical methods to be replaced with more complex models to analyze such data, with machine learning (ML) being on the top of the used methodologies (Figure 3). The main task of ML is to analyze existing data, and on their basis, construct a predictive model, which is then used for the evaluation of new examples (Mitchell, 2014).

Machine learning tools can be categorized into unsupervised (clustering methods) and supervised (regression, classifier analysis) learning. Both of these types are used in the search for new drugs, depending on the wanted outcome. ML is useful in all steps of the new drug discovery pipeline, especially in tasks where vast amounts of data need to be analyzed. It helps in the identification of new potential ligands in the VS procedure, generation sets of new potentially active compounds [deep learning, (DL)], optimization of compound physicochemical and ADMET properties, and detection of compound interactions with off-targets (Carpenter et al., 2018; Chen et al., 2018). ML is also useful in pre- and clinical development for cell response classification after drug intake (Vamathevan et al., 2019), as well as after the introduction of the drug to the market, e.g., for analyzing and monitoring the drug efficiency and possible side effects (Dimitri and Lió, 2017; Gao et al., 2017).

In an exemplary ML-based study to search for new anti-mycobacterial compounds, Prakash and colleagues built a database of compounds with known antitubercular effect, divided into three activity classes. Then, the compounds were clustered according to their chemical structure and four clusters acting on different targets were selected for further analysis. Cluster numbered 10 consisted of compounds, e.g., aminohydrazones, iso-nicotinoyl hydrazones, and iso-nicotinohydrazides that inhibit KatG and 2-trans-enoyl-acyl carrier protein reductase (InhA). Cluster 57 included pyrrole derivatives and azole antifungals, which interact with CYP51 isozymes. The next cluster contained oxazolidinones, which bind DNA gyrase, and 2-benzylthiopydidine-4-carbithioamide derivatives, which targets are not known. The last cluster contained pyridobenzoxaine derivatives of LFX and nitroquinolones. Hologram QSAR (HQSAR) allowed the search for fragments of molecules contributing to particular compound activity and to detect moieties that discriminated against active and inactive compounds. Individual active motifs found *via* this procedure were fused, and new bioactive motifs were proposed. Furthermore, they verified the effectiveness of new motifs by comparing them to compounds in the previously constructed dataset. One of the created motifs was found in already existing drugs: RMP, rifabutin, cirpofloxacin (COX), and ofloxacin (OX; Prakash and Ghosh, 2006).

Another similar approach used in drug design is searching for molecular patterns in other known drugs. In one of the early studies, researchers built their computational model by combining four linear equations and then apply it to screen the compounds found in Merck and Sigma-Aldrich catalogs. They selected 18 new compounds for microbial tests, which have not worked in their favor despite careful preparation of the model. ML models at that time were far from ideal. However, the study was able to pick four compounds that already have been experimentally checked for inhibiting *M. tuberculosis* growth, e.g., LZD, paromomycin, reserpine, and trifluoperazine from 5,000 compounds in the database, as well as compounds with new structures not found in currently used drugs treating TB (García-García et al., 2005). Some researchers are leaning toward Bayesian models since they are better suited for global QSAR analysis, can manage more data, and the results are easier to interpret and reproduce. The good performance of this model was proven by a study in which out of 44 antituberculars only six (13,6%) were assigned to the wrong group based on chemical structure (Prathipati et al., 2008). In a similar study, Ekins and colleagues found through Bayesian modeling drugs that have not been yet experimentally verified against *M. tuberculosis* but scored high, e.g., sertaconazole, clofarabine, tioconazole, amodiaquine, quinaldine blue, atorvastatin, montelukast, daunorubicin, 4'-methoxychalcone, inosine, hieracin, iridin, harmane, and irigenol (Ekins and Freundlich, 2011).

Some researchers guided by the principles of polypharmacology are moving away from the “one target-one hit” model and looking into drugs that can potentially inhibit multiple targets (Zhang et al., 2016). The reasoning of this approach lies in the fact that treatment for TB already consists of multiple antimicrobials administered for a very long time (over 6months for drug-susceptible *M. tuberculosis*; Tiberi et al., 2018a). Therefore drugs inhibiting multiple targets would significantly simplify treatment. There is also the possibility that adequate multi-target compounds will be more effective against drug-resistant TB and will not lead to the emergence of resistance as fast as one-target drugs do. Following this reasoning, Speck-Planche et al. (2012) created a model for mt-QSAR (multiple target QSAR). The difference between this method and QSAR was in the training dataset, which was constructed based on compounds active against all six proteins GyrA, GyrB, InhA, Ag85C, PS, and PD. Through combined VS, QSAR, and structure-based pharmacophore models, researchers found initial hits against InhA, GlmU, and DapB. Next, they added other known drug targets to create possible multi-target drugs (Janardhan et al., 2017). In another study focusing on phytochemicals through VS, researchers found four compounds amentoflavone, carpaine, 13-bromo-tiliacorinine and 2-nortiliacorinine, that bind with high affinity to many *M. tuberculosis* proteins, like Ask, DdIA, PanC, TrpB, AroF, NadE, AtpE, RibH, RpIE, and RpsE (Kumar et al., 2019). All results described above need yet to be confirmed by *in vitro* studies.

Since ML is used as a tool for finding new compounds through the mining of chemical databases, a number of databases gathering information on compound structure and bioactivity have been constructed. An example of such a database is a Collaborative Drug Discovery (CDD, Burlingame, CA), which now consists of more than 200,000 molecules (Hohman et al., 2009; Ekins et al., 2010). Since its construction, CDD was used in many important TB projects like the EU-funded New Medicines 4 Tuberculosis (NM4TB) initiative (Ekins et al., 2011). Other popular databases of bioactive molecules are ChEMBL (which now contains almost 2 million distinct compounds, with over 16 million biological activities annotated) and PDSP with ~10,000 compound affinities toward different targets gathered (Besnard et al., 2012; Gaulton et al., 2012).

## Consideration of Drug Resistance

The principal molecular basis for mycobacterial diversity and drug resistance are single-nucleotide polymorphisms (SNPs). SNPs occur in the genome as a result of replication errors or erroneous DNA repair. *M. tuberculosis* lacks horizontal gene transfer trough mobile genetic elements such as plasmids or transposons. The comparison of the genomes of various *M. tuberculosis* strains revealed their similarity at over 99% (Namouchi et al., 2012).

Many mutations encoding drug resistance are located in the direct drug targets of the proteins, e.g., *rpo*B (RMP; Telenti et al., 1993), *emb*CAB operon (EMB; Mikusová et al., 1995; Telenti et al., 1997), *rrs* (KAN; Georghiou et al., 2012), *gyr*A, *gyr*B (fluoroquinolones, FQ; Takiff et al., 1994). However, drug resistance mutations are also associated with other *loci*. For example, in the case of INH, mutations occur in a seemingly unrelated *ahp*C gene encoding alkyl hydroperoxidase It turns out that AhpC takes over the role of KatG, which is a catalase-peroxidase responsible for the transformation of INH from pro-drug to effective drug, in protecting the genome from oxygen-induced damage. The overexpression of AhpC significantly slows down the production of KatG. Thus, less INH is activated, and cells can survive (Sherman et al., 1999).

Mutations occurring in drug targets can negatively affect proteins metabolic activity, resulting in a deficit of cell fitness. Bacterial cells compensate through compensatory mutations. Mutants carrying compensatory mutations are characterized by lower fitness costs associated with drug resistance. The best-known example of drug-resistant mutations causing fitness cost is RpoB (RMP resistance; Gagneux et al., 2006). Compensatory mutations were found in RpoB and other proteins of RNA polymerase complex, RpoA, and RpoC (Gagneux et al., 2006; Comas et al., 2012; de Vos et al., 2013).

### DNA Sequencing

The principal identification of drug resistance sources is based on the cultivation of bacteria with antibiotics until drug-resistant variants appear (Takiff et al., 1994; Telenti et al., 1997). The genome sequences of drug-resistant clones are sequenced and screened for mutations (Telenti et al., 1997). This approach was used to identify drug-resistant mutations for major antitubercular drugs like EMB (Telenti et al., 1997) and FQ (Takiff et al., 1994). Notably, the amount of drug-resistant variants that can be detected in such studies is limited. As a high-throughput method, TraSH can be used to identify genes that do not sustain insertion among the pool of mutants (Sassetti et al., 2003; van Opijnen et al., 2009). Researchers tested the sensitivity of 69 morphotype mutants of *M. smegmatis* to one of the commonly used antibiotics – ampicillin to identify cell envelope genes associated with B-lactam resistance. After receiving four sensitive mutants, the transposon insertion sites were mapped (Viswanathan et al., 2017).

### Transcriptomics and Genetic Modification of Bacteria

Changes in gene expression profiles studied through RNA-seq allow understanding of antibiotics’ effect on *M. tuberculosis* physiology regarding tolerance mechanisms and drug resistance (Jain et al., 2016; Briffotaux et al., 2019). For example, increased the expression of the *efpA* gene encoding the efflux pump from the MFS family (major facilitator superfamily) and the *iniA* gene encoding one of the putative components of the efflux pump, after treatment with INH, may indicate that microorganisms are acquiring resistance (Briffotaux et al., 2019). Exposition of *M. tuberculosis* to antibiotics results in the overexpression of genes encoding DNA repair proteins (Gorna et al., 2010), e.g., *dinX* after RMP treatment (Boshoff et al., 2004), *ssb* after CM treatment (Fu and Shinnick, 2007), *ada*, *alkA* after treatment LFX (Boshoff et al., 2004) or *xthA* after treatment with OX (Boshoff et al., 2004) and many others.

The induction of efflux pumps can be visualized with reporter systems. Jain et al. (2016) investigated the gene expression profile in the presence of a subinhibitory concentration of INH. The addition of INH to *M. tuberculosis* during the logarithmic growth phase caused a CFU decrease of 2–3 logs. Mycobacterial cells that survived the treatment were characterized by an increased expression of specific genes, which indicate the formation of persister cells. These gene promoters were fused to a gene encoding the red fluorescent protein to create a reporter system for persisters.

### Bioinformatic Predictions

Genome-wide association studies (GWAS) identify drug resistance sources after the drugs are already on the market. GWAS utilize genomic DNA sequencing data and statistics to study the association between gene variants across the population and the phenotypic traits, for example, variants of genes giving rise to drug-resistant strains (Power et al., 2017). Importantly, GWAS studies allow the identification of rare drug resistance variants or low-level drug resistance variants. GWAS of Farhat et al. (2013) study showed that potentially noncoding regions of the genome, like promoters of genes, contribute to drug resistance. The same team has confirmed noncoding regions associated with drug resistance for INH, EMB, and PZA. However, their effect on drug MIC was smaller than the effect of coding region mutations (Farhat et al., 2019). In subsequent studies, Coll et al. (2018) used GWAS to identify drug-resistance associated mutations in 6465 clinical strains. Most of the mutations and loci they found were well-known, but some of them were new, including *loci* in *foli*C, *ubi*A, *thy*X-*hsd*S.1, *thy*A, *alr*, *ald*, and *dfr*A-*thy*A. They also found mutations in *eth*A and *thy*X promoters that may contribute to resistance emergence. GWAS is also used for studying characteristics of populations of *M. tuberculosis*. Oppong et al. (2019) through GWAS analysis, examined whether phylogenetic lineage background impacts drug resistance and found that particular drug resistance specific *loci* occur only in selected lineages.

The development of -omic technologies and computational power allowed the development of biological network models. These models are based on systemic biology, which combines knowledge about organisms on all organizational levels. The models take into account all the complex interactions and mechanisms occurring in the cells. There are different types of such networks, e.g., transcription factors-binding, PIP networks, metabolic interaction networks, genetic interaction networks, and others (Zhu et al., 2007; Chandra et al., 2011; Chung et al., 2013).

*In silico* analyses like PIP are applicable in predicting drug resistance patterns through building networks based on the STRING database and defining proteins that are drug targets and possible components of resistance emergence. Through this research, the concept of “co-target” was created. Co-target is a protein used simultaneously as a primary bacterial growth inhibitor to stop the emergence of resistance by affecting proteins in the resistance emergence pathway. These co-targets can be proteins associated with SOS response (RecA, RuvA, and LexA), involved in HGT (SecA1 and SecA2) or metabolism of drugs inside the cell like cytochromes and degrading enzymes (Cyp135, Erm37), but also proteins involved in the transportation of drugs out of the cell through efflux pumps (PstB; Raman and Chandra, 2008). Besides, PIP networks allow for learning more about the effects of antibiotics on the cells by finding what pathways are set in motion when antibiotics kill bacteria (Kohanski et al., 2010).

Identification of rare drug resistance mutations can also be made through ML. Based on 1983 *M. tuberculosis* isolates, Yang et al. (2018) developed ML models for four first-line drugs – INH, RMP, EMB, PZA, and several second-line drugs that analyze WGS data. The models increased the sensitivity of the detection of drug resistance when compared with previous studies. For EMB and RMP, the sensitivity increased to 97% (*p* < 0.01), and for COX and multi-drug resistant TB, it increased to 96%. The INH had the lowest results, with only a 2–4% increase. In another study, Deelder et al. (2019) utilized WGS data to compare GWAS and their ML model. Both methods reached similar results, but GWAS was slightly more accurate for CM, CS, and KAN.

In the best scenario, mutations associated with drug-resistance are confirmed through obtaining genetic mutants and observing the resulting drug-resistant phenotype. Another approach to confirm that a mutation is linked to drug resistance is through *in silico* docking. *In silico* docking is a useful tool for predicting the roles of mutations causing drug resistance. It can be done by analyzing interactions between the selected drug and protein, the wild-type, and the mutated variant (Nachappa et al., 2020).

## Entering the Preclinical Stage

Researchers thoroughly check compounds with the potential to become drugs during the early discovery stage. Regardless of their usefulness from a biological point of view, novel compounds should have form facilitating administration. They have to go through tests of solubility, stability, and reactivity (Strovel et al., 2004; Hughes et al., 2011). If they fail to obtain satisfactory parameters, they are discarded. If they reach a satisfactory biological safety level and are practical, they proceed to the preclinical drug discovery stage. Preclinical studies are a checkpoint before human administration, and thus they are vital and carried with much caution.

Most of the compounds going through a hit to lead, and lead optimization, do not enter the preclinical stage. If we count all of the structures considered in chemical databases for screening, millions of compounds do not make it to the preclinical stage. Therefore the preclinical stage is often less costly than the early discovery stage. Up to our knowledge, there are no recent estimates on how many compounds go from the preclinical stage to the clinical phase. A common statement found in the articles is that out of 5,000 compounds entering the preclinical stage, five make it to the clinical stage, and then one makes it to the market (Kraljevic et al., 2004). Taken how many compounds undergo tests, the models for early drug discovery need to efficiently investigate broad biological consequences in a short time and at a low-cost.

## Conclusion

The growing demand for discovering new antibiotics stimulates the constant development of research methods that broaden the knowledge about biological processes occurring in bacterial cells undergoing chemotherapy. Understanding these processes enables more effective identification of antitubercular compounds. Thanks to the -omics technologies, these compounds can now be safer and less prone to the generation of drug resistance. When combined, the -omics technologies allow us to gain a more holistic view of drug utility.

## Author Contributions

AM and JD designed the manuscript. AM, LŻ, EL, FG, AK, SP, DZ, and JD wrote the manuscript. All authors contributed to the article and approved the submitted version.

### Conflict of Interest

The authors declare that the research was conducted in the absence of any commercial or financial relationships that could be construed as a potential conflict of interest.
